# Investigating the Impact of Climate Warming on Phenology of Aphid Pests in China Using Long-Term Historical Data

**DOI:** 10.3390/insects11030167

**Published:** 2020-03-05

**Authors:** Yangxue Wu, Junjie Li, Huanhuan Liu, Gexia Qiao, Xiaolei Huang

**Affiliations:** 1 State Key Laboratory of Ecological Pest Control for Fujian and Taiwan Crops, College of Plant Protection, Fujian Agriculture and Forestry University, Fuzhou 350002, China; 1180203024@fafu.edu.cn (Y.W.); 15090629031@163.com (J.L.); layhyhh@sina.com (H.L.); 2 Key Laboratory of Zoological Systematics and Evolution, Institute of Zoology, Chinese Academy of Sciences, Beijing 100101, China; qiaogx@ioz.ac.cn

**Keywords:** global warming, life cycle, migration, overwintering, temperature

## Abstract

Global climate warming has significant influence on individual development, population dynamics, and geographical distribution of many organisms, which has drawn much attention in recent years. As a large group of poikilotherms, insects whose life activities are closely linked with ambient temperature are supposed to be influenced by global warming. In order to test the consistency or difference of the effects of long-term climate warming on phytophagous insect pests in different geographical environments, this study collected historical data on the occurrence and population dynamics of three aphid pests (*Myzus persicae*, *Aphis gossypii,* and *Sitobion avenae*) in China, and systematically explored their phenological responses. We found that, during a period of about 60 years, in general, the first occurrence dates and the first migration dates of the three aphids almost moved earlier, while the end of the occurrence and the last migration dates were slightly delayed. However, these responses also represented geographical variation at a local scale. Basically, our results showed that the occurrence and migration seasons of these three aphid pests have been prolonged along with climate warming. This study based on historical literature data provides empirical evidence and valuable implications for understanding the impact of climate warming on insect pests and future management strategies.

## 1. Introduction

As one of the main characteristics of global climate change, global warming has been widely concerned. The Intergovernmental Panel on Climate Change (IPCC) points out that the mean global temperature has increased by 0.72 °C during the period of 1880 to 2012 with an average rise rate of 0.12 °C/10 years [1]. Global climate change has a significant impact on geographical distribution, population dynamics, and phenology of many organisms [2]. In recent years, the impact of climate warming on insects has become a research hotspot. As poikilotherms, the life activities of insects such as growth and development, survival, reproduction and migration depend closely on ambient temperature, that is, they may inevitably be affected by the global warming [3,4,5,6]. Therefore, how to cope with climate warming becomes a key issue for individual survival and population development of insects [7].

Previous studies have shown that climate warming can accelerate the growth and development of insects, leading to earlier occurrence and longer life cycle [8,9,10]. For example, the breeding time of spruce beetles *Dendroctonus rufipennis* in the northwestern North America has been cut in half with the increasing temperature [11]. Interestingly, it is observed that higher temperature would speed up the completion of the insect reproductive cycle so as to produce more generations in different insects such as butterflies, bees, dragonflies and damselflies, flies and beetles [12]. From the data of the British Butterfly Monitoring Schemes, Roy and Sparks found that most British butterflies first appeared earlier and the period of their average flight became longer due to the rising temperature of spring and summer in central England between 1976 and 1998 [13]. A similar result was also revealed for the adult insects of 37 Odonata species in the Netherlands [14].

Aphids with a small body size and short life cycle are sensitive to the change in ambient temperature [15]. They are model species that can better reflect the relationship between insect biology and climate fluctuation [16], having a wide range of host plants and are widely distributed in most parts of the world [17]. Many aphids are seriously harmful plant pests by stunting plant growth, inducing plant galls, causing deformation of leaves, buds and flowers, and transmitting plant virus diseases [18,19]. In addition, many aphid species have specific life characters, such as migration, to reduce competition and avoid unfavorable environments and natural enemies [20], which are often significantly affected by climatic factors [21,22,23]. Therefore, aphids may show different life cycle strategies in different environments and regions. Aphids with holocycle induce sexual aphids due to temperature and other factors, and produce fertilized eggs to overwinter [24], while aphids with anholocycle can reproduce parthenogenetically throughout the year and move between multiple hosts without obvious wintering [25]. For example, the life cycle of the green peach aphid *Myzus persicae* varies depending on the temperature. In colder temperate regions, the reproductive mode of *M. persicae* is generally cyclical parthenogenetic, in which the overwintering eggs are usually hatched around March and early April on primary host plants (winter hosts), and the aphid populations generally reach peak migrations from May to June with fastest reproduction and most harmfulness on secondary host plants (summer hosts), and at the end of the fall they move back to winter hosts to mate and lay eggs [26]. While in warmer regions, the *M. persicae* have cyclical parthenogenesis, as well as obligate parthenogenesis. The migration of aphid pests often causes sudden outbreak in local areas and great economic losses to agricultural and forestry production, which is a major challenge for agroforestry. Therefore, exploring changes of the occurrence of aphids in a long historical period can provide information for forecasting and comprehensive control of pests.

Several studies have reported that climate change, especially temperature change, has significant influence on the occurrence and growth of aphids. For instance, the higher the temperature spring had, the earlier the time when cotton aphids began to hatch, and the earlier the time they took to harm cotton was [27]. Due to climate warming, the first flight time of the British spruce aphid *Elatobium abietinum* occurred ahead and its flight season was prolonged between 1966 and 2006 [28]. It was also found that faster spring temperature warming could cause migration of the soybean aphids to advance [29]. In Poland, male aphids appeared earlier due to the warming climate from 2005 to 2014 [30]. Based on suction-trap data from 1964 to 2014, the Rothamsted Insect Survey (RIS) found that under the influence of global warming, the first migratory time of 55 aphid species in the UK was earlier and the average migratory season of most species was significantly extended, although different species showed varied response patterns [16]. In addition, the first appearance of the nymphs of juniper aphid *Cinara juniperi* in the temperate areas of Poland emerged earlier as local spring temperature increased [31]. Moreover, warm winter weather may also cause changes in aphid life cycles [32]. In southern Poland, during the warmer early winter of 2013–2014, female adult aphids of *Stomaphis* spp. successfully lived for four months longer than usual [33], while female adults of the genus usually died in harsh severe conditions after laying eggs [34].

These indicate that aphids may adapt to climate change by adjusting their own growth and development and migration time. However, considering that spatial heterogeneity of climate warming may exist across geographical scales, and it is not yet known whether phenological responses to climate warming are consistent or different among more species, investigating these issues with more aphid pests and from different geographical regions is urgent.

China, with vast territory, complex terrain, and diverse climate types, is also significantly affected by global warming. However, the long-term effects of climate warming on aphid pests are largely unknown due to the lack of long-term data on population monitoring. To conquer this obstacle, historical data from the literature may provide valuable information for understanding this issue [35,36,37]. Therefore, our study collected historical data on the occurrence and population dynamics of three important agricultural pests of aphids, *Myzus persicae*, *Aphis gossypii,* and *Sitobion avenae*. All three species can harm lots of economic plants and crops [38,39], among them *M. persicae* and *A. gossypii* are rated as TOP 10 Arthropod Pests by the Centre for Agriculture and Biosciences International (CABI) in the State of the World’s Plants 2017 report [40]. Based on the long-term data we collected, we investigated the influence of long-term climate change on these aphids by analyzing changes of several life cycle parameters.

## 2. Materials and Methods

### 2.1. Phenological Data of Aphids

The phenological data of aphids in this study were extracted and compiled from historical literature, most of which were retrieved from the CNKI database (http://www.cnki.net), the most extensive and comprehensive database of Chinese periodicals and magazines [41]. First, the species names of the three aphids were used as subject words and respectively searched by using the method of subject word retrieval. Then, the relevant literature reporting the occurrences and geographical distributions of the three aphids in various regions of China were consulted from January 1951 to December 2017 (see Supplementary Table S1). A total of 201 related articles were collected, of which 84 were related to *M. persicae*, 80 were related to *A. gossypii*, and 37 were related to *S. avenae*. The specific time and geographic information on life cycle parameters were extracted and a database was established.

The collected data were organized based on four life cycle parameters, including the first occurrence date, the first migration date, the end of occurrence, and the end of migration. We defined the hatching time of overwintering eggs as the first occurrence date, the time of mating and spawning on the winter host as the end of occurrence, the first time of starting to migrate as the first migration date, and the time of migration back to winter host as the end of migration. There were some vague time descriptions of these parameters in some literature, such as “the beginning of the month”, “the end of the month”, “the first (or last) ten days”. Therefore, we specifically quantified such time information without specific dates. For example, the description about the beginning of a month was set as the first day of that month while the end of a month was set as the last day of that month, and the first, middle, and last ten days of a month were set as 5th, 15th, and 25th of that month, respectively.

The collected data of *A. gossypii* was mainly concentrated in Xinjiang of northwestern China, as it is the main cotton producing region with suitable conditions for cotton production [42], and therefore, the most historical literature of *A. gossypii* have been reported data from this region. This gave us an opportunity to test spatial heterogeneity of the impact of climate warming at a local geographical scale. All data collection sites reported in the collected literature of the three aphid pests (*M. persicae*, *A. gossypii,* and *S. avenae*) were georeferenced into geographical maps by using the ArcGIS 10.2 (ESRI, Inc., Redlands, CA, USA), which were presented in the Supplementary Figure S1.

### 2.2. Meteorological Data

We obtained the annual average temperatures of China from 1959 to 2017. Temperature records were retrieved from Chinese meteorological websites (http://data.cma.cn/), the China Environmental Bulletins, and the China Climate Bulletins. Related to the *A. gossypii* data, we also calculated the annual average temperature of representative areas in Xinjiang from 1951 to 2013. Given that the east–west Tianshan Mountains crosses the central part of Xinjiang, the climate difference between the southern and northern Xinjiang is obvious, which would have a certain impact on the life history of aphids. Therefore, based on our collected geographical distributions of *A. gossypii* in Xinjiang, we selected six meteorological stations in southern Xinjiang (including Turpan, Kumul, Korla, Aksu, Kashgar, and Shache) and two sites in northern Xinjiang (including Usu and Caijiahu), and calculated the annual average temperatures of the above eight sites, separately. Then, the average of the southern six stations and the average of the two northern sites was used to represent the annual average temperatures of the southern and the northern Xinjiang, respectively. In addition, considering that aphid phenology parameters (first and last occurrence or migration) are more affected by spring and winter temperatures, we also calculated the mean, maximum, and minimum temperatures of spring (March–May) and winter (December–February) in China, as well as representative areas of Xinjiang mentioned above from 1951 to 2014. A simple linear regression method was used to calculate the overall change rates of temperature in China, as well as the southern and the northern Xinjiang in about the past 60 years and analyze the trend and process of temperature change.

### 2.3. Statistical Analysis

We analyzed the phenological responses of the aphids by plotting changes of the life cycle parameters mentioned above (the beginning of occurrence, the end of occurrence, the first migration, and the last migration). The occurrence year was taken as the X-axis, and the change of days for each life cycle parameter was taken as the Y-axis. Linear regression analysis was implemented to establish regression equations, and the trends of the four life cycle parameters in the time series were analyzed, respectively.

To conveniently quantify the “change of days”, for each aphid species and each life cycle parameter, we first calculated the differences (“number of days”) between the dates of occurrence or migration records in our dataset and 1 January, and then the averages of the numbers of days were calculated. By adopting the average as a reference with a value of zero on the Y-axis, the change of days of occurrence or migration were plotted and used to determine whether the trends of aphid emergence or migration were earlier or later in a long term.

We first carried out a normal test for the quantified data sets, which showed that most data sets were under normal distributions, except for two data sets of the beginning and the end of the occurrence of *M. persicae*. For the two data sets not following normal distribution, we implemented model fitting to select the most suitable model, which indicated that the fitting degree and significance of the linear model were optimal. In order to improve the accuracy of further analyses, these two data sets were first normalized. The linear regression equation was obtained from regression analysis which was used to investigate the relationship between the year series and the change of days with climate warming. The Pearson coefficient was used to test the correlation between time and the date of occurrence or migration. All analyses were carried out using SPSS for Windows version 24.0 (SPSS Inc., Chicago, IL, USA).

## 3. Results

### 3.1. Temperature Changes in China and Xinjiang in Recent Decades

**Figure 1:**
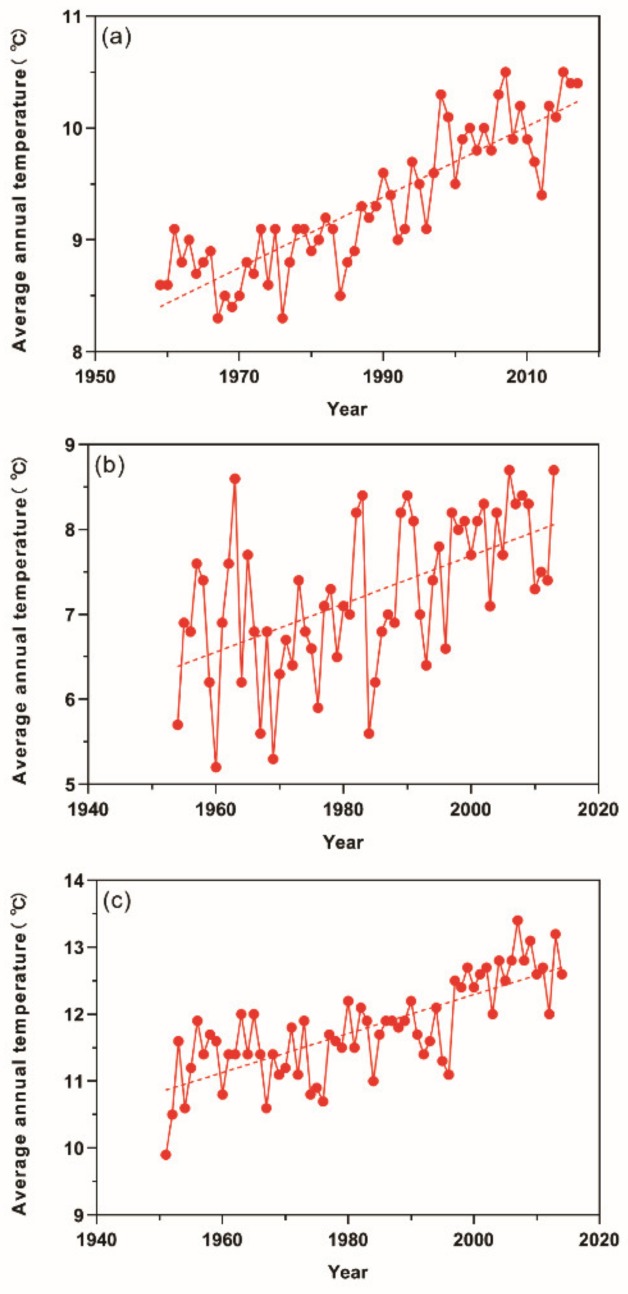
The change of annual average temperature (AAT) in China, as well as Xinjiang in recent decades. The dot represents AAT in a specific year and the dashed line shows the trend of temperature change. (**a**) China as a whole; (**b**) northern Xinjiang; (**c**) southern Xinjiang.

Since 1959, the annual average temperature (referred as AAT hereafter) in China presented an increasing trend with fluctuations. Through linear regression calculation, the AAT increased about 0.0316 ± SE 0.002 °C (*p* < 0.01) each year (Figure 1a). The AAT in China in 1959 was 8.6 °C while that in 2017 was 10.4 °C. A change of 1.8 °C happened in the past about 60 years. From 1954 to 2013, the AAT in northern Xinjiang showed an upward trend (Figure 1b), from 5.7 °C in 1954 to 8.7 °C in 2013. The linear regression analysis indicated that the AAT in this region increased by about 0.0281 ± SE 0.006 °C year^−1^ (*p* < 0.01). In addition, from 1951 to 2013, the change of AAT in southern Xinjiang was similar with an increasing trend (Figure 1c), within which region the AAT increased from 9.9 °C in 1951 to 13.2 °C in 2013. Moreover, a temperature change rate of about 0.0292 ± SE 0.003 °C year^−1^ (*p* < 0.01) was revealed by the linear regression calculation. It could also be seen that while the temperatures in both northern and southern Xinjiang were on the rise in the case of climate warming, the initial AAT, as well as the increasing rate in southern Xinjiang were higher than that in northern Xinjiang.

**Figure 2:**
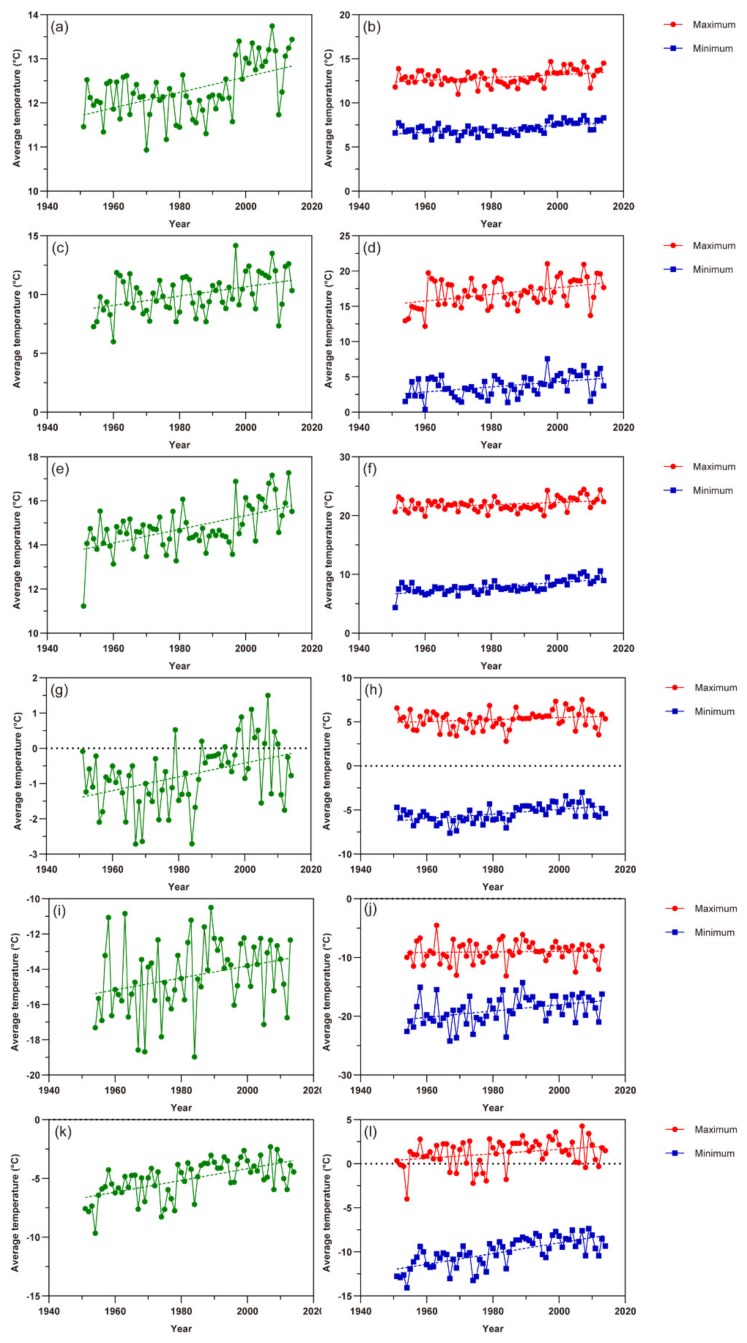
The change of the average temperature of spring (SAT) and winter (WAT) in China, as well as Xinjiang in recent decades (left column), and the change of the highest (SHT) and lowest (SLT) temperatures of spring (SHT, SLT) and winter (WHT, WLT) (right column). The dashed line with color shows the trend of temperature change. (**a**) SAT in China as a whole; (**b**) SHT and SLT in China as a whole; (**c**) SAT in northern Xinjiang; (**d**) SHT and SLT in northern Xinjiang; (**e**) SAT in southern Xinjiang; (**f**) SHT and SLT in southern Xinjiang; (**g**) WAT in China as a whole; (**h**) WHT and WLT in China as a whole; (**i**) WAT in northern Xinjiang; (**j**) WHT and WLT in northern Xinjiang; (**k**) WAT in southern Xinjiang; (**l**) WHT and WLT in southern Xinjiang.

In the past 60 years, the average temperatures of the spring (March–May) and winter (December–February) in China as a whole, as well as in Xinjiang have also been increasing in general, both in terms of average temperature and the maximum or minimum temperature in each season (Figure 2). Through linear regression calculation, the average temperature of spring (referred as SAT hereafter) and winter (referred as WAT hereafter) in China increased about 0.0176 ± SE 0.004 °C (*p* < 0.01) and 0.0195 ± SE 0.006 °C (*p* < 0.01) each year, respectively (Figure 2a,g). The highest (SHT) and lowest (SLT) temperatures of spring rose by 0.0164 ± SE 0.005 °C (*p* < 0.01) and 0.0195 ± SE 0.004 °C (*p* < 0.01) per year, respectively (Figure 2b). Additionally, the change rate of the highest (WHT) and lowest (WLT) temperatures of winter were about 0.0112 ± SE 0.007 °C (*p* = 0.1004) and 0.0261 ± SE 0.005 °C (*p* < 0.01) yearly (Figure 2h). Similar rising trends of temperature parameters could also be seen in both northern and southern Xinjiang.

### 3.2. The Response of the First Migration

**Figure 3:**
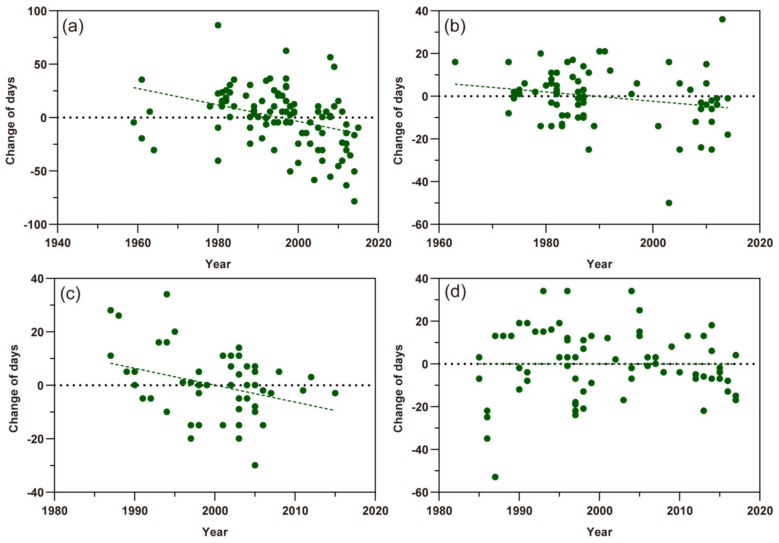
Scatter plots of the first migration times of three aphid pests. The dots represent different records and the dashed lines show the trends of the first flight migration. (**a**) *Myzus persicae*; (**b**) *Sitobion avenae*; (**c**) *Aphis gossypii* in northern Xinjiang; (**d**) *A. gossypii* in southern Xinjiang.

Based on scatter plots of the first migration times of the three aphid species (Figure 3), except the *A. gossypii* in southern Xinjiang, the scatter points of change of first migration of *M. persicae*, *S. avenae*, and *A. gossypii* in northern Xinjiang showed a tendency to lower and more negative values in the Y-axis with the year series, indicating that the date of the first migration of these aphids appeared earlier. Among them, the first migration date of *M. persicae* and *S. avenae* was advanced by 0.770 ± SE 0.202 days year^−1^ (t = −3.682, *p*< 0.01) and 0.216 ± SE 0.103 days year^−1^ (t = −2.092, *p* < 0.05), respectively (Figure 3a,b). The first migration date of *A. gossypii* in northern Xinjiang was with a change rate of 0.632 ± SE 0.250 days (t = −2.527, *p* < 0.05) earlier per year (Figure 3c). However, this life cycle parameter of *A. gossypii* in southern Xinjiang was almost not changed (−0.0024 ± SE 0.186 days per year; t = −0.013, *p* = 0.990) (Figure 3d).

### 3.3. The Response of the Last Migration

**Figure 4:**
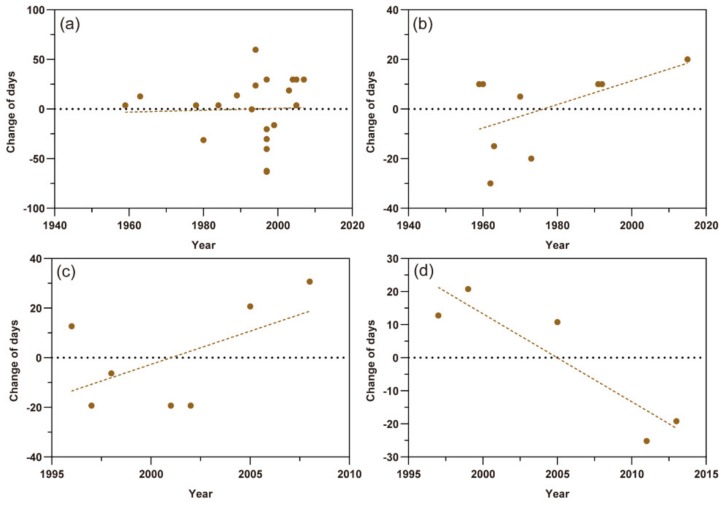
Scatter plots of the last migration times of three aphid pests. The dots represent different records and the dashed lines show the trends of the last flight migration. (**a**) *Myzus persicae*; (**b**) *Sitobion avenae*; (**c**) *Aphis gossypii* in northern Xinjiang; (**d**) *A. gossypii* in southern Xinjiang.

For the last migration, the scatter plots of *M. persicae*, *S. avenae,* and *A. gossypii* in northern Xinjiang showed an upward trend more or less in the time series, which predicted that the times of their last migration were delayed. The changes of last migration time of *M. persicae* and *S. avenae* varied by 0.096 ± SE 0.565 days (t = 0.170, *p* = 0.867) per year and 0.475 ± SE 0.285 days (t = 1.667, *p* = 0.140) per year, respectively (Figure 4a,b). The last migrations of the *A. gossypii* in northern and southern Xinjiang showed different trends by 2.681 ± SE 1.789 (t = 1.499, *p* = 0.194) days per year and −2.660 ± SE 0.709 days (t = −3.751, *p* < 0.05) per year, respectively (Figure 4c,d). However, this contrast may be still uncertain due to few data points.

### 3.4. The Response of the Beginning of Occurrence

**Figure 5:**
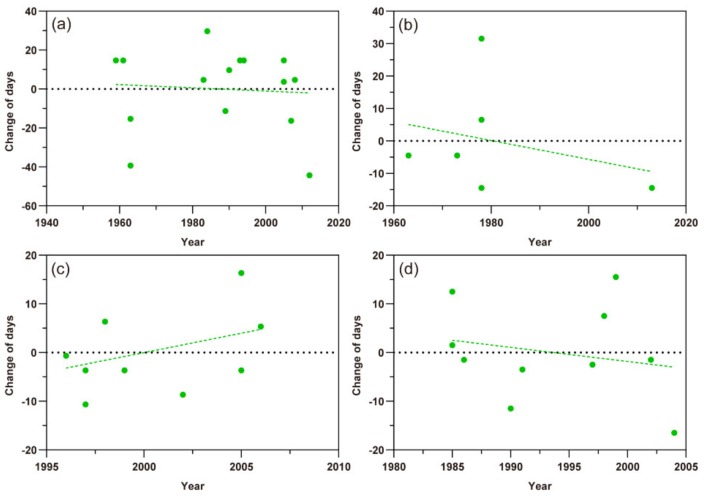
Scatter plots of the first occurrence dates of three aphid pests. The dots represent different records and the dashed lines show the trends of the first occurrence. (**a**) *Myzus persicae*; (**b**) *Sitobion avenae*; (**c**) *Aphis gossypii* in northern Xinjiang; (**d**) *A. gossypii* in southern Xinjiang.

*M. persicae* and *A. gossypii* had more data records while *S. avenae* had fewer data about the first occurrence defined by overwintering egg hatching. The scatter plots of Figure 4 showed that the distribution of occurrence dates of the *M. persicae*, *S. avenae,* and *A. gossypii* in southern Xinjiang followed a downward trend, indicating that the incubation time of their wintering eggs became earlier. However, the current data collection indicated that the first occurrence of *A. gossypii* in the northern Xinjiang was delayed (Figure 5c). The earliest hatching times of overwintering eggs of *M. persicae* and *S. avenae* varied by −0.009 ± SE 0.014 (t = −0.684, *p* = 0.506) and −0.290 ± SE 0.489 days (t = −0.594, *p* = 0.584) per year, respectively (Figure 5a,b). The hatching time of cotton aphid overwintering eggs in southern and northern Xinjiang changed by –0.290 ± SE 0.477 days per year (t = −0.608, *p* = 0.560) and 0.796 ± SE 0.643 days every year (t = 1.237, *p* = 0.247), respectively (Figure 5c,d).

### 3.5. The Response of the End of Occurrence

**Figure 6:**
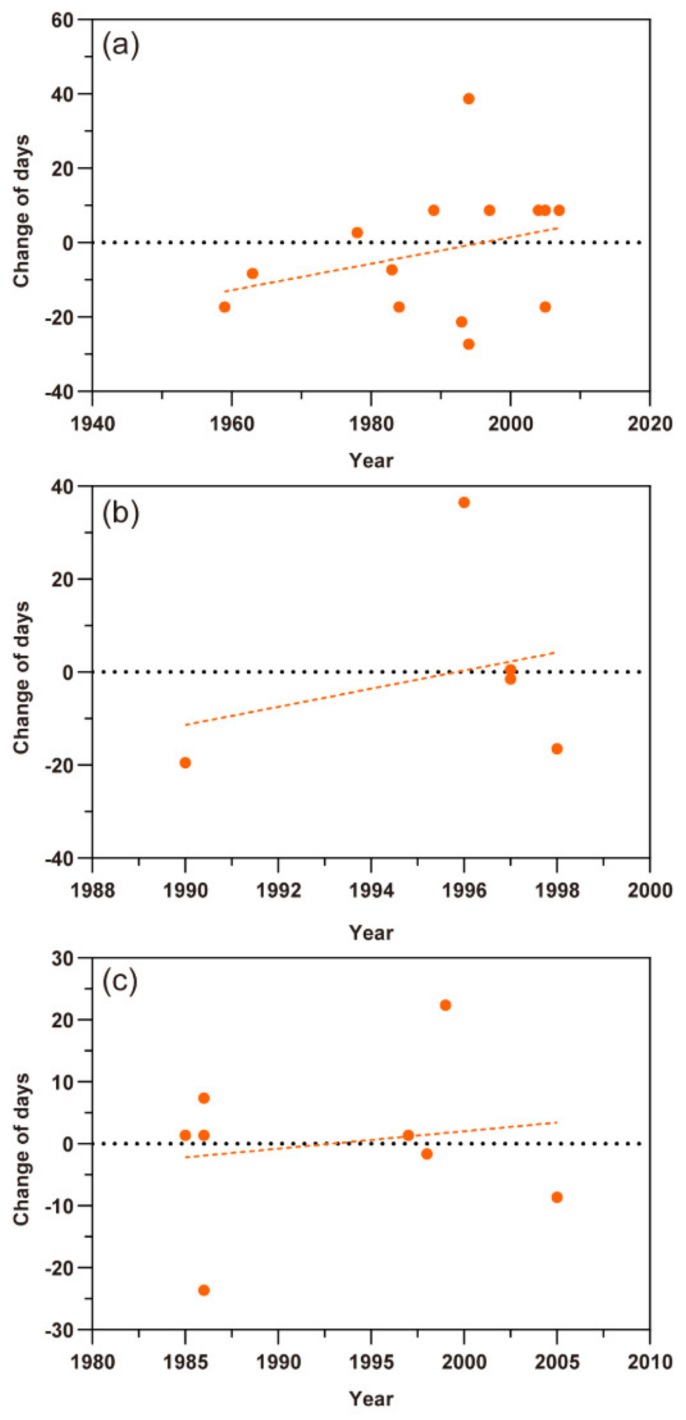
Scatter plots of the last occurrence dates of three aphid pests. The dots represent different records and the dashed lines show the trends of the last occurrence. (**a**) *Myzus persicae*; (**b**) *Aphis gossypii* in northern Xinjiang; (**c**) *A. gossypii* in southern Xinjiang.

In general, scatter plots of the last occurrence dates of *M. persicae,* as well as *A. gossypii* in Xinjiang showed an upward shift, which indicated that the times for mating and spawning on winter hosts were slightly delayed. The oviposition time of the green peach aphid advanced by 0.018 ± SE 0.017 (t = 1.053, *p* = 0.313) (Figure 6a), while this life cycle parameter of *A. gossypii* in northern and southern Xinjiang advanced by 1.949 ± SE 3.263 days each year (t = 0.597, *p* = 0.582) and 0.281 ± SE 0.670 days each year (t = 0.419, *p* = 0.689), respectively (Figure 6b,c). As too little information about the end of occurrence of *S. avenae* had been reported in historical literature, we discarded analysis of it for this species.

## 4. Discussion

Our analyses indicated that, during a period of about 60 years, the annual average temperatures of China as a whole, as well as the Xinjiang region have shown rising trends, and the average temperatures in the spring and winter showed similar trends. Under the condition of climate warming, in general, the first migration times of the three aphids (i.e., *M. persicae*, *S. avenae,* and *A. gossypii*) occurred earlier. This may be due to the warming climate that leads to earlier spring and faster reaching of the minimum threshold of population size aphids needed to migrate. Similarly, the temperature increase could make earlier incubation of overwintering eggs of the three aphids, therefore the first occurrence dates were also advanced. We also found that the last migration dates mostly showed slight delay. The frequent occurrence of warm winter may make the aphids that originally rely on spawning for overwintering continue to grow in parthenogenesis on secondary hosts, instead of flying back to winter hosts [33,43,44]. This may also cause a slight delay of the end of occurrence defined by mating and spawning on winter hosts, as shown by our data. Basically, these responses indicate prolonged occurrence and migration seasons of these aphid pests throughout the time series. However, data accumulated so far have indicated that the effects of climate change are much more complex than the simple linear response to the increasing average temperature, and there may be differences between different regions [45,46,47]. In fact, our data in northern and southern Xinjiang may also indicate spatial heterogeneity of the effect of climate warming at a local scale. For instance, the average temperature in southern Xinjiang was higher than that in northern Xinjiang. These may lead to earlier occurrence and migration times of aphids in southern Xinjiang than in northern Xinjiang. The average first migration time of *A. gossypii* in southern Xinjiang was around mid-April, while the average first migration time in northern Xinjiang was around mid-late April. The data for the other two species (*M. persicae* and *S. avenae*) in Xinjiang region also supported that, the further north the distribution sites were, basically the later the first migration time would be in a same year, as was also found in a previous study in the UK [16]. In addition, the long-term trends of last migration and the first occurrence of the cotton aphid showed different patterns between the northern and southern Xinjiang. There is a possibility that this contrast may be a real reflection of spatial heterogeneity of the impact of climate warming. However, uncertainty still exists due to few data accumulation for specific life cycle parameters.

Considering that aphid phenology is temperature-dependent, the weather anomaly in a specific year may introduce “noise” (i.e., unusual values for the life cycle parameters) in a long-term data set. For example, in 1996, the first migration of *A. gossypii* in southern Xinjiang was reported on 15 June, later than the average. According to literature records, the temperature was unstable in May of that year, heavy rain and low temperature appeared, which may delay the migration of aphids. However, in general, the first migration of the cotton aphid in southern Xinjiang is slightly ahead of time. Similarly, severe cold damage occurred in Heilongjiang province of the northeastern China in 1980, and the first migration time of *M. persicae* was postponed to mid-late July. Differences in geography or host plant may also affect aphid phenology. For example, in the Huangzhong county of Qinghai province with an altitude of about 2800 m, the first migration of *M. persicae* was later than the average. This is understandable considering the temperature decreases along with an increase of the altitude. Moreover, the establishment of greenhouses close to cities may affect the overwintering strategy of aphids and therefore introduce specific unusual records. There was one case of influence of greenhouse on the first migration of aphids in our dataset: *M. persicae* on peach trees in the greenhouse in Hami region of Xinjiang showed much earlier migration than the average.

The extension of occurrence and migration seasons of these aphid pests mean a possible increase of damage time, which will be more harmful for the growth and development of crops. Our results can provide theoretical guidance for the future prediction and prevention of aphid pests. In addition, it should be noted that the time of aphid damage has been advanced caused by temperature increase, which may lead to mismatch of phenological synchronicity between aphids and host plants or natural enemies [48,49]. If the insects hatch early, but the host plants it feeds on do not germinate at the same time, then the young individuals cannot feed normally and die [50]. As an alternative response, insects may turn to new plants for food in order to survive, resulting in the expansion of host plant range. As described in a previous study, the host plants that the willow psyllid *Cacopsylla groenlandica* feeds on has expanded from just one type of willow to four along a climatic gradient [51]. The advanced occurrence of aphids may also affect the original phenological synchronization between aphids and parasitic wasps, leading to the decrease of parasitic rate [52]. Equally important is that rising temperatures could make aphids more prone to parthenogenesis and spread to higher altitudes and latitudes, potentially increasing the range of crop damage [32,53]. These are challenges to monitoring and controlling aphid pests in the future.

Understanding the long-term impact of climate warming on insect pests is of great significance in both science and application. However, evidence on this issue has still been limited [35,36,37], mainly due to the lack of long-term dataset. The present paper provides another empirical study by collecting nearly 60 years dataset on population dynamics of three notorious aphid pests. It should be noted that due to the ambiguity of time information provided by some literature, certain uncertainty could occur when quantifying the time data. Therefore, to minimize the impact of uncertainty in such historical literature data, careful data collection, standardization, and analyses are indispensable. Cautious analyses based on such historical dataset can still contribute valuable implications for uncovering the effects of climate warming on aphid occurrence and migration in general. This study will provide a reference for future studies to explore related issues in more organism groups and geographical regions. In the future, a more detailed investigation of the long-term impact of climate warming on aphids and other insect pests, as well as standard monitoring network by implementing, for example, suction traps, are very needed in China.

## 5. Conclusions

Based on the collection and analyses of historical data of three aphid pests (*Myzus persicae*, *Aphis gossypii,* and *Sitobion avenae*) in China, this study revealed the long-term effects of climate warming on phenology of these aphids across large temporal and spatial scales. Results showed that the annual average temperatures and average temperatures in the spring and winter of China as a whole as well as the Xinjiang region have shown rising trends over the past 60 years. With climate warming, in general, the first occurrence and migration dates of these aphids have almost moved earlier, while the end of occurrence and the last migration dates have been slightly delayed, indicating prolonged occurrence and migration seasons. The data of *A. gossypii* in northern and southern Xinjiang may also indicate spatial heterogeneity of phonological responses to climate warming at a local scale. The present paper proves the value of historical literature data for uncovering long-term impact of climate warming on insect phenology, and provides important implications for future prediction and control of insect pests.

## Supplementary Materials

The following are available online at https://www.mdpi.com/2075-4450/11/3/167/s1. Table S1: A list of source references for retrieving the phenological data of the three aphids used in this study. Figure S1: The data collection sites of the three aphids used in this study. (**a**) *Myzus persicae*; (**b**) *S. avenae*; (**c**) *A. gossypii* (blue, northern Xinjiang; cyan, southern Xinjiang); (**d**) All three aphid species.
