# Supplementary figures and images for: Investigating the Impact of Climate Warming on Phenology of Aphid Pests in China Using Long-Term Historical Data
Source: Insects. 2020 Mar 5;11(3):167. doi: 10.3390/insects11030167 (PMC7143611; doi:10.3390/insects11030167)

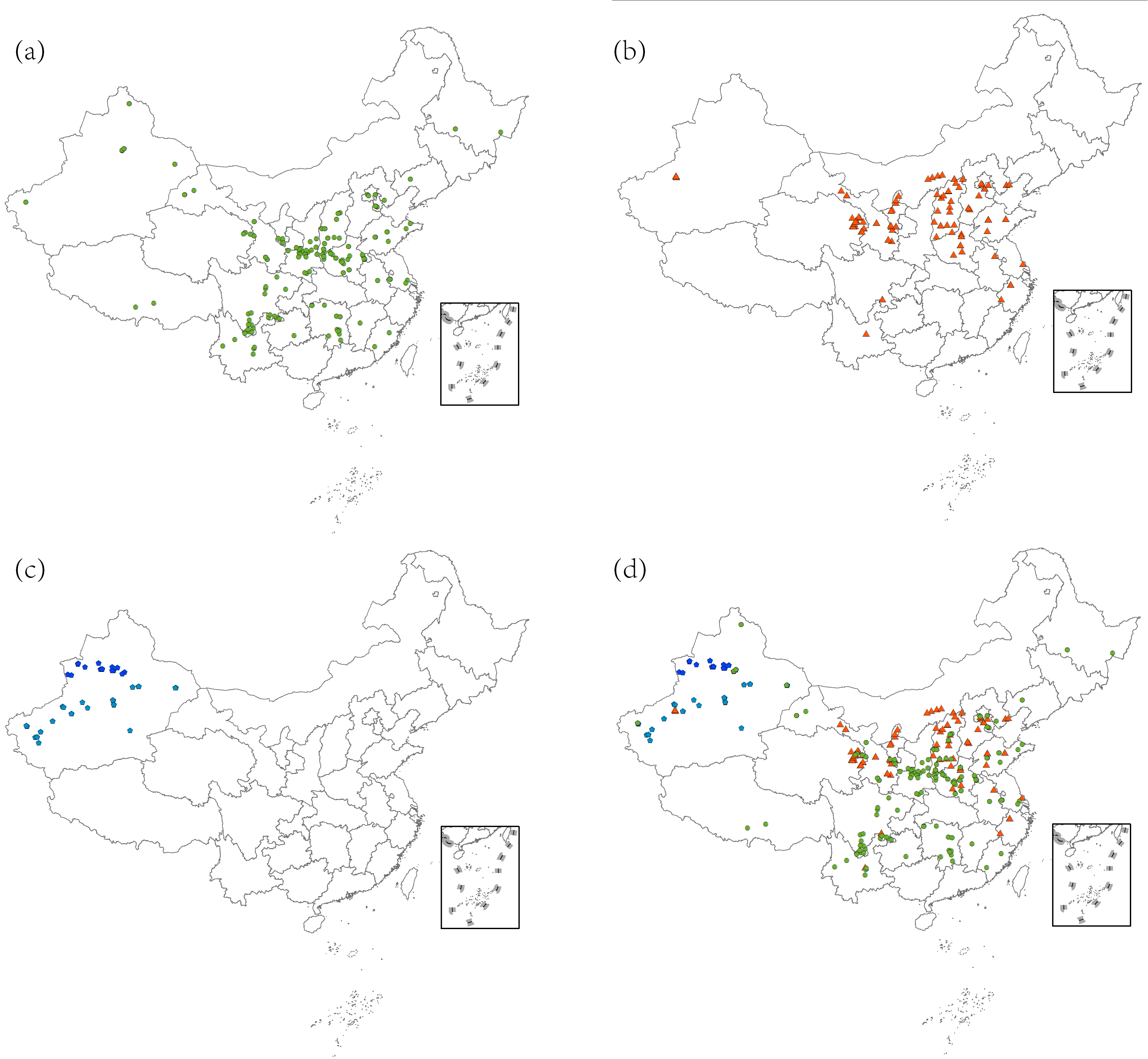

Supplement: Supplementary file 1 [file insects-11-00167-s001.zip › supplementary files/Figure S1_data collection sites.tif]
